# High Neutrophil-to-Lymphocyte Ratio and Platelet-to-Lymphocyte Ratio Are Associated With Sarcopenia Risk in Hospitalized Renal Cell Carcinoma Patients

**DOI:** 10.3389/fonc.2021.736640

**Published:** 2021-10-25

**Authors:** Qiang Hu, Weipu Mao, Tiange Wu, Zhipeng Xu, Junjie Yu, Can Wang, Saisai Chen, Shuqiu Chen, Bin Xu, Yueshuang Xu, Ming Chen

**Affiliations:** ^1^ Department of Urology, Zhongda Hospital, Southeast University, Nanjing, China; ^2^ Surgical Research Center, Institute of Urology, Southeast University Medical School, Nanjing, China; ^3^ Department of Clinical Research, Southeast University Medical School, Nanjing, China; ^4^ Department of Pathology, School of Medicine, Southeast University, Nanjing, China; ^5^ Department of Urology, Nanjing Lishui District People’s Hospital, Zhongda Hospital Lishui Branch, Southeast University, Nanjing, China

**Keywords:** renal cell carcinoma, sarcopenia, neutrophil–lymphocyte ratio, platelet–lymphocyte ratio, inflammation

## Abstract

**Purpose:**

This study aimed i) to identify the best cutoff points of neutrophil–lymphocyte ratio (NLR) and platelet–lymphocyte ratio (PLR) that predict sarcopenia and ii) to illustrate the association between sarcopenia risk and NLR or PLR in renal cell carcinoma (RCC) patients undergoing laparoscopic partial or radical nephrectomy.

**Methods:**

A total of 343 RCC patients who underwent laparoscopic partial or radical nephrectomy between 2014 and 2019 were enrolled in our study. Sarcopenia was assessed by lumbar skeletal muscle index (SMI). Receiver operating characteristic (ROC) curve was used to identify the best cutoff point of NLR or PLR to predict sarcopenia risk. Univariate and multivariate logistic regression and dose–response analysis curves of restricted cubic spline function were conducted to assess the relationship between sarcopenia and NLR or PLR.

**Results:**

The best cutoff points of NLR >2.88 or PLR >135.63 were confirmed by the ROC curve to predict sarcopenia risk. Dose–response curves showed that the risk of sarcopenia increased with raising NLR and PLR. Patients with NLR >2.88 or PLR >135.63 had a higher sarcopenia risk than those in the NLR ≤2.8 or PLR ≤135.63 group, respectively. By adjusting for all variables, we found that patients with NLR >2.88 and PLR >135.63 had 149% and 85% higher risk to develop sarcopenia, respectively, than those with NLR ≤2.8 (aOR = 2.49; 95% CI = 1.56–3.98; *p* < 0.001) or PLR ≤135.63 (aOR = 1.85; 95% CI = 1.16–2.95; *p* = 0.010).

**Conclusion:**

In RCC patients receiving laparoscopic partial or radical nephrectomy, NLR and PLR, which were biomarkers of systemic inflammation, were associated with sarcopenia risk.

## Introduction

Renal cell carcinoma (RCC) is a common malignance with a morbidity of 2%–3% in systemic cancers (1). Clear cell renal cell carcinoma as the most common subtype accounts for 80%–85% of RCC cases. With its increasing morbidity, 170,000 patients diagnosed with RCC died in 2018 worldwide, and the mortality is about 2.7% (2). Patients diagnosed with RCC at an early stage can be effectively treated by radical or partial nephrectomy, resulting in a 5-year survival rate up to 93%. However, more than 30% of patients were progressed to advanced RCC at the first diagnosis, and 10%–20% early-stage RCC patients would experience recurrence after the treatments. Due to regional and distant metastases, the 5-year survival rate of advanced RCC patients decreases to 67% (3). Although molecular targeted therapy and immunotherapy, with or without cytoreductive surgery, remain the widely used treatments (4), it is still a clinical challenge to prolong the survival of advanced RCC patients.

In addition to the early diagnosis, there are multiple other factors that affect the prognosis of RCC patients, including tumor size, pathological stage, and other biochemical indicators. Studies have confirmed that the development of sarcopenia is a risk factor for poor survival time of patients with colon or liver cancer (5, 6). Our previous study also showed that sarcopenia is a factor which can affect the poor prognosis of OS and CSS in RCC patients (7). The potential reasons might be the reduced treatment tolerance, increased toxic reaction of chemotherapeutic drugs, prolonged length of hospital stay, and increased postoperative complications. Thus, the prediction and early diagnosis of sarcopenia is crucial for the prognosis of cancer patients.

Sarcopenia, a progressive skeletal muscle disorder, usually increases the likelihood of adverse outcomes, such as fractures, falls, physical disability, and even mortality (8, 9). In 2018, sarcopenia was defined as the detection of low muscle mass and decreased muscle function by the European Working Group on Sarcopenia in Older People (EWGSOP) (10). Studies have shown that there was a complex pathophysiologic mechanism for the development of sarcopenia, including the downregulation of anabolic hormones such as growth hormone and increased myocyte apoptosis and circulating inflammatory cytokines (11–14). The inflammatory cytokines produced by tumor cells and leukocytes, including tumor necrosis factor alpha (TNF-α) and interleukin-1, interleukin-6, and interleukin-8 (IL-1, IL-6, and IL-8), are demonstrated to be the major drivers of cachexia. These cytokines affect a variety of tissues, which include skeletal muscle, brain, liver, etc. (15). Among them, TNF-α can activate the NF-κB pathway, followed by ubiquitin-mediated proteasome catabolism of muscle protein (16).

It was demonstrated that the increased number of inflammatory cells was associated with the development of sarcopenia (17, 18), which is a risk factor of poor survival in cancer patients. However, there is still a lack of study to confirm the relationship between inflammation and sarcopenia in Chinese RCC patients. Here, we investigated the clinical data of RCC patients in our hospital to analyze the relationship between inflammation and sarcopenia in Chinese RCC patients.

## Patients and Methods

### Patient Selection

A clinical database of urinary cancers has been collected by the Department of Urology of Zhongda Hospital. In this study, we retrospectively selected 354 RCC cases receiving laparoscopic partial or radical nephrectomy in Zhongda Hospital from 2014 to 2019. This research conformed to the criteria outlined in the Helsinki Declaration and was ethically approved by the Ethics Committee and Institutional Review Board of Zhongda Hospital (ZDKYSB077). In addition, all the patients and their relatives who were enrolled in this study signed the informed consent form.

The inclusion criteria were as follows: a) age ≥18 years, b) diagnosis with RCC, and c) experienced laparoscopic partial or radical nephrectomy. The exclusion criteria were as follows: a) diagnosis with more than one cancer type (*n* = 6); b) other disease-related conditions that significantly affect survival, including liver cirrhosis and seizure history (*n* = 3); and c) incomplete or missing follow-up data (*n* = 2). According to these criteria, 11 patients were excluded and a total of 343 patients were enrolled in the final study cohort.

### Data Collection and Follow-Up

All related clinical data of the patients were collected from their electronic medical records in Zhongda Hospital, including gender (male and female), age (≤65 and >65 years), body mass index (BMI) (<25 and ≥25 kg/m^2^), hypertension (no and yes), diabetes (no and yes), cardiovascular disease (no and yes), smoking (no and yes), surgery type (partial nephrectomy and radical nephrectomy), laterality (left and right), American Joint Committee on Cancer (AJCC) stage (I, II, III, and IV), T stage (T1, T2, T3, and T4), N stage (N0 and N1), M stage (M0 and M1), Fuhrman grade (I, II, III and IV), and sarcopenic (no and yes). Preoperational blood samples were collected for further analysis. The follow-up of discharged patients receiving nephrectomy was performed on an outpatient or telephone basis. Survival time was defined as the time ranging from the end of surgery to the end of follow-up or death.

### Anthropometric Measurements and Sarcopenia Risk Evaluation

Body weight (kg) and height (m) were measured using an electronic weighing scale and stadiometer. Body mass index (BMI, kg/m^2^) was obtained accordingly.

**Figure 1:**
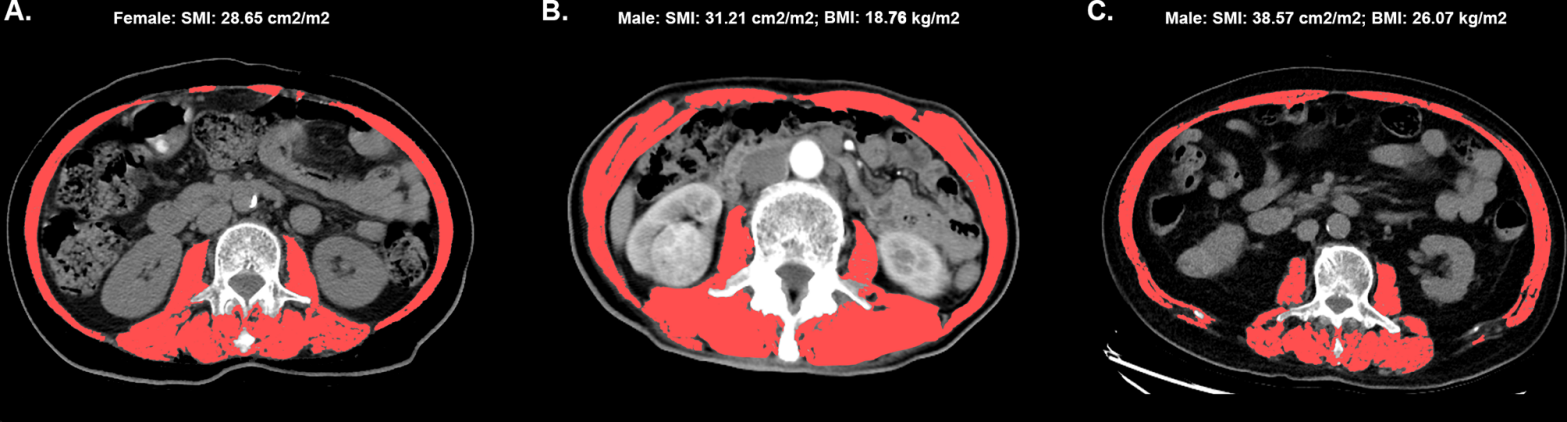
The cross-sectional image of the third lumbar vertebra, and the skeletal muscle is highlighted in red. The labels **(A–C)** represents three different patient images, **(A)** The image of female patient with SMI 28.65 cm^2^/m^2^; **(B)** The image of male patient with SMI 31.21 cm^2^/m^2^ and BMI 18.76 kg/m^2^; **(C)** The image of male patient with SMI 38.57cm^2^/m^2^ and BMI 26.07 kg/m^2^.

The lumbar skeletal muscle index (SMI) measured with American GE Revolution CT scanner before surgery was the indicator for evaluating sarcopenia. The CT images were provided by trained radiologists, and the skeletal muscle area (SMA, cm^2^) was measured according to the total muscle area, including bilateral psoas, paraspinal, internal oblique, external oblique, rectus abdominis, and transversus abdominis muscles. Thus, SMI (SMA (cm^2^)/((height(m))^2^) could be calculated accordingly (**Figure 1**).

According to the criteria from the international consensus group, sarcopenia was diagnosed as follows: SMI <41 cm^2^/m^2^ in female or SMI <4 3cm^2^/m^2^ in male, with BMI <25 kg/m^2^ or SMI <53 cm^2^/m^2^ and BMI ≥25 kg/m^2^ (19).

### Biochemical Analysis

All venous blood samples were collected by trained professionals, and an identical automated system was used to concentrate blood cells and to analyze biochemical components.

### Statistical Analysis

SPSS software (version 24.0) and RStudio software (version 1.2.5033) were used for all analysis in this study and *P <*0.05 was set as statistically significant.

Categorical variables were expressed as *n* (%) and analyzed by chi-square test, while parametric variables were analyzed by Student’s *t*-test and expressed as mean ± standard deviation. The best cutoff point of NLR or PLR to predict sarcopenia risk was assessed by the sensitivity, specificity, and area under the receiver operating characteristic (ROC) curve. The significance level was set at 5%. Univariate and multivariate logistic regressions were used to assess the association between sarcopenia and NLR or PLR. Restricted cubic spline function was also used to characterize the dose–response relationships between sarcopenia and NLR or PLR by adjusting variables.

## Results

**Figure 2:**
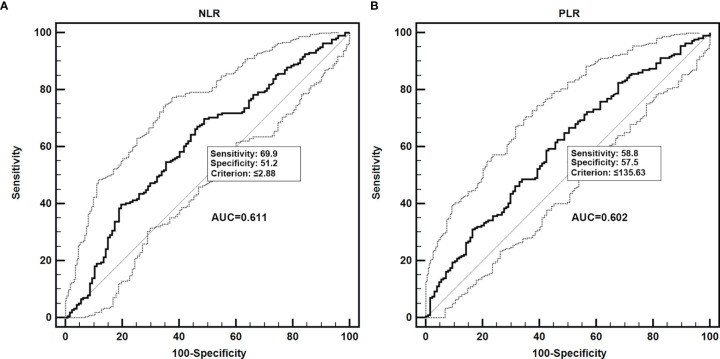
Receiver operating characteristic (ROC) curves to assess the best predictive values of NLR and PLR for identifying the risk of sarcopenia in patients with renal cancer carcinoma. NLR, neutrophil–lymphocyte ratio; PLR, platelet–lymphocyte ratio. **(A)** ROC curve of NLR; **(B)** ROC curve of PLR.

The ROC curve showed that the best cutoff point to define the sarcopenia risk was NLR >2.88 (AUC = 0.611, 95% CI = 0.557–0.663, *p* = 0.0004, with sensitivity of 69.9% and specificity of 51.2%) or PLR >135.63 (AUC = 0.602, 95% CI = 0.548–0.654, *p* = 0.0012, with sensitivity of 58.8% and specificity of 57.5%) (**Figure 2**).

The clinical data of 343 patients enrolled in this study are all shown in **Table 1**. According to the best cutoff point from the analysis of the AUC in **Figure 2**, all patients were divided into two groups, respectively: NLR ≤2.8 group and NLR >2.88 group or PLR ≤135.63 group and PLR >135.63 group. From the data, we did not observe a significant difference for age, gender, smoking, medical history (including diabetes, hypertension, and cardiovascular diseases), laterality and histological type. However, patients in the NLR >2.88 group had a higher sarcopenia risk than those in the NLR ≤2.8 group (49.6% *vs*. 29.2%, *p* < 0.001). Moreover, a similar result was observed in the PLR group. Patients in the PLR >135.63 group had a higher sarcopenia risk than those in the PLR ≤135.63 group (45.1% vs. 29.8%, *p* = 0.004) (**Table 1**).

**Table 1:** Clinical characteristics of the patients according to NLR and PLR.

Characteristics		NLR			PLR		
	Total	≤2.88	>2.88	*P*-value	≤135.63	>135.63	*P*-value
	Patients	*N* = 212	*N* = 131		*N* = 181	*N* = 162	
	*N* = 343	Median (IQR)	Median (IQR)		Median (IQR)	Median (IQR)	
Age, years, mean ± SD	57.47 ± 12.56	56.97 ± 11.87	58.27 ± 13.61	0.351	58.12 ± 11.47	56.74 ± 13.68	0.310
Age categorized, years				0.921			0.699
≤65	255 (74.3)	158 (74.5)	97 (74.0)		133 (73.5)	122 (75.3)	
>65	88 (25.7)	54 (25.5)	34 (26.0)		48 (26.5)	40 (24.7)	
Gender				0.693			0.953
Male	226 (65.9)	138 (65.1)	88 (67.2)		119 (65.7)	107 (66.0)	
Female	117 (34.1)	74 (34.9)	43 (32.8)		62 (34.3)	55 (34.0)	
BMI, kg/m^2^, mean ± SD	24.69 ± 3.62	25.08 ± 3.54	24.07 ± 3.67	0.012	25.10 ± 3.65	24.23 ± 3.54	0.026
BMI categorized, kg/m^2^				0.233			0.037
<25	185 (53.9)	109 (51.4)	76 (58.0)		88 (48.6)	97 (59.9)	
≥25	158 (46.1)	103 (48.6)	55 (42.0)		93 (51.4)	65 (40.1)	
Hypertension				0.814			0.010
No	191 (55.7)	117 (55.2)	74 (56.5)		89 (49.2)	102 (63.0)	
Yes	152 (44.3)	95 (44.8)	57 (43.5)		92 (50.8)	60 (37.0)	
Diabetes				0.546			0.763
No	288 (84.0)	180 (84.9)	108 (82.4)		153 (84.5)	135 (83.3)	
Yes	55 (16.0)	32 (15.1)	23 (17.6)		28 (15.5)	27 (16.7)	
Cardiovascular diseases				0.387			0.920
No	300 (87.5)	188 (88.7)	112 (85.5)		158 (87.3)	142 (87.7)	
Yes	43 (12.5)	24 (11.3)	19 (14.5)		23 (12.7)	20 (12.3)	
Smoking				0.818			0.371
No	286 (83.4)	176 (83.0)	110 (84.0)		154 (85.1)	132 (81.5)	
Yes	57 (16.6)	36 (17.0)	21 (16.0)		27 (14.9)	30 (18.5)	
ASA score				0.289			0.225
1–2	325 (94.8)	203 (95.8)	122 (93.1)		169 (93.4)	156 (96.3)	
3–4	18 (5.2)	9 (4.2)	9 (6.9)		12 (6.6)	6 (3.7)	
Clavien–Dindo complications				0.246			0.386
None	330 (96.2)	207 (97.6)	123 (93.9)		177 (97.8)	153 (94.4)	
I	6 (1.7)	2 (0.9)	4 (3.1)		2 (1.1)	4 (2.5)	
II	6 (1.7)	3 (1.4)	3 (2.3)		2 (1.1)	4 (2.5)	
III	1 (0.3)	0 (0.0)	1 (0.8)		0 (0.0)	1 (0.6)	
Surgery type				<0.001			<0.001
Partial nephrectomy	187 (54.5)	135 (63.7)	52 (39.7)		117 (64.6)	70 (43.2)	
Radical nephrectomy	156 (45.5)	77 (36.3)	79 (60.3)		64 (35.4)	92 (56.8)	
Laterality				0.462			0.212
Left	171 (49.9)	109 (51.4)	62 (47.3)		96 (53.0)	75 (46.3)	
Right	172 (50.1)	103 (48.6)	69 (52.7)		85 (47.0)	87 (53.7)	
Histological type				0.057			0.132
Clear cell carcinoma	269 (78.4)	170 (80.2)	99 (75.6)		147 (81.2)	122 (75.3)	
Papillary cell carcinoma	17 (5.0)	12 (5.7)	5 (3.8)		11 (6.1)	6 (3.7)	
Chromogenic carcinoma	16 (4.7)	12 (5.7)	4 (3.1)		5 (2.8)	11 (6.8)	
Others	41 (12.0)	18 (8.5)	23 (17.6)		18 (9.9)	23 (14.2)	
AJCC stage				0.002			0.222
I	256 (74.6)	173 (81.6)	83 (63.4)		143 (79.0)	113 (69.8)	
II	19 (5.5)	10 (4.7)	9 (6.9)		7 (3.9)	12 (7.4)	
III	45 (13.1)	20 (9.4)	25 (19.1)		20 (11.0)	25 (15.4)	
IV	23 (6.7)	9 (4.2)	14 (10.7)		11 (6.1)	12 (7.4)	
T stage				0.001			0.161
T1	260 (75.8)	176 (83.0)	84 (64.1)		145 (80.1)	115 (71.0)	
T2	23 (6.7)	12 (5.7)	11 (8.4)		8 (4.4)	15 (9.3)	
T3	51 (14.9)	20 (9.4)	31 (23.7)		23 (12.7)	28 (17.3)	
T4	9 (2.6)	4 (1.9)	5 (3.8)		5 (2.8)	4 (2.5)	
N stage				0.547			0.626
N0	330 (96.2)	205 (96.7)	125 (95.4)		175 (96.7)	155 (95.7)	
N1	13 (3.8)	7 (3.3)	6 (4.6)		6 (3.3)	7 (4.3)	
M stage				0.040			0.459
M0	327 (95.3)	206 (97.2)	121 (92.4)		174 (96.1)	153 (94.4)	
M1	16 (4.7)	6 (2.8)	10 (7.6)		7 (3.9)	9 (5.6)	
Fuhrman grade				0.052			0.011
I	55 (16.0)	40 (18.9)	15 (11.5)		33 (18.2)	22 (13.6)	
II	216 (63.0)	136 (64.2)	80 (61.1)		121 (66.9)	95 (58.6)	
III	64 (18.7)	33 (15.6)	31 (23.7)		26 (14.4)	38 (23.5)	
IV	8 (2.3)	3 (1.4)	5 (3.8)		1 (0.6)	7 (4.3)	
Sarcopenic				<0.001			0.004
No	216 (63.0)	150 (70.8)	66 (50.4)		127 (70.2)	89 (54.9)	
Yes	127 (37.0)	62 (29.2)	65 (49.6)		54 (29.8)	73 (45.1)	
Urea nitrogen	6.62 ± 4.89	6.36 ± 5.48	7.04 ± 3.71	0.211	6.62 ± 6.00	6.61 ± 3.25	0.980
Creatinine	119.58 ± 96.05	109.69 ± 91.61	135.60 ± 101.14	0.015	119.23 ± 105.10	119.98 ± 85.12	0.943
Uric acid	262.83 ± 101.36	250.65 ± 96.75	282.54 ± 105.85	0.004	263.90 ± 93.43	261.64 ± 109.83	0.837
Hemoglobin	133.14 ± 20.34	137.43 ± 16.62	126.19 ± 23.68	<0.001	136.81 ± 17.71	129.04 ± 22.27	<0.001
Albumin	41.08 ± 5.02	41.75 ± 4.66	39.98 ± 5.39	0.001	41.41 ± 4.97	40.70 ± 5.06	0.187

Percentages may not total 100 because of rounding.

IQR, interquartile range; BMI, body mass index; NLR, neutrophil-to-lymphocyte ratio; PLR, platelet-to-lymphocyte ratio.

**Figure 3:**
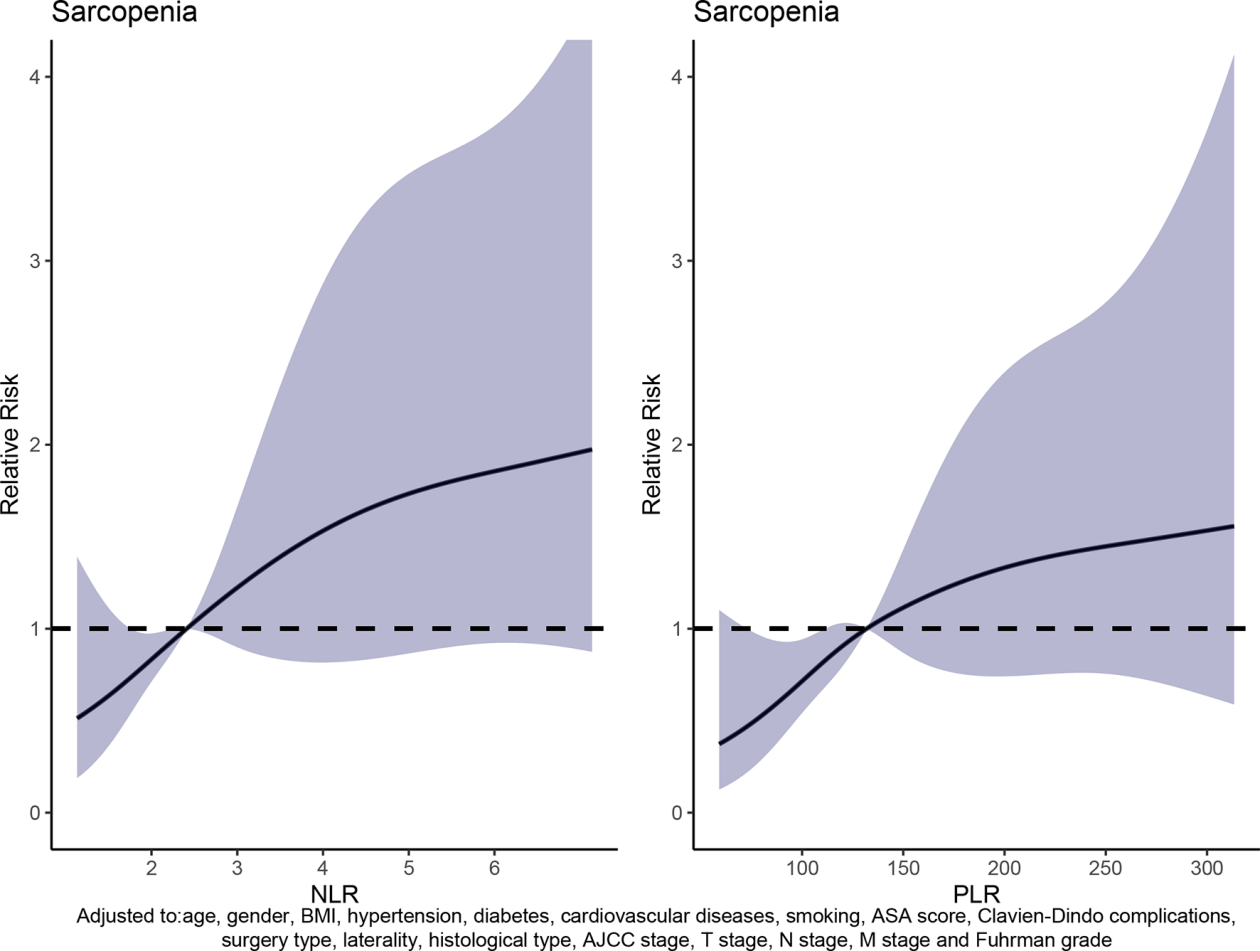
Relationship between NLR and PLR with sarcopenia risk.

The dose–response curve was obtained by adjusting covariates including age, gender, BMI, hypertension, diabetes, cardiovascular diseases, smoking, surgery type, laterality, AJCC stage, T stage, N stage, M stage, and Fuhrman grade. It also showed that patients had a greater risk to develop sarcopenia as NLR or PLR increased (**Figure 3** and **Table 2**). In addition, the optimal NLR and PLR were 2.40 and 131.91, respectively, when the OR was 1.

**Table 2:** Weighted odds ratio and 95% confidence intervals of sarcopenia by levels of NLR or PLR.

**NLR**	**2**	**3**	**4**	**5**	**6**
**Overall (2.43)**	**0.84 (0.72–0.97)**	**1.22 (0.90–1.67)**	**1.53 (0.82–2.87)**	**1.73 (0.87–3.45)**	**1.85 (0.92–3.73)**
PLR	100	150	200	250	300
Overall (131.91)	0.71 (0.54–0.94)	1.11 (0.87–1.42)	1.33 (0.74–2.39)	1.45 (0.76–2.77)	1.53 (0.63–3.73)

The following covariates were adjusted: age, gender, BMI, hypertension, diabetes, cardiovascular diseases, smoking, ASA score, Clavien–Dindo complications, surgery type, laterality, histological type, AJCC stage, T stage, N stage, M stage, and Fuhrman grade.

BMI, body mass index; AJCC, American Joint Committee on Cancer; NLR, neutrophil-to-lymphocyte ratio; PLR, platelet-to-lymphocyte ratio.

To further evaluate the relationships between sarcopenia risk and NLR or PLR level, we used logistic regression to analyze the results. It showed that NLR or PLR level was an independent risk factor for the development of sarcopenia no matter whether in the basic model, core model, or extended model. In detail, as the extended model analyzed by adjusting for all variables, we found that patients with NLR >2.88 had a 149% higher risk of sarcopenia than those with NLR ≤2.8 (aOR = 2.49; 95% CI = 1.56–3.98; *p* < 0.001), and patients with PLR >135.63 had a 85% higher risk to develop sarcopenia than those with PLR ≤135.63 (aOR = 1.85; 95% CI = 1.16–2.95; *p* = 0.01) (**Table 3**).

**Table 3:** Relative risk of sarcopenia was calculated according to NLR or PLR levels^a^.

Characteristic	*N*	Basic model		Core model		Extended model	
		aOR (95% CI)	*P*-value	aOR (95% CI)	*P*-value	aOR (95% CI)	*P*-value
NLR			**0.020**		**0.010**		**0.031**
≤2.88	212	1.00		1.00		1.00	
>2.88	131	**2.38 (1.52–3.75)**	**<0.001**	**2.48 (1.56–3.95)**	**<0.001**	**2.49 (1.56–3.98)**	**<0.001**
PLR			**0.001**		**<0.001**		**0.006**
≤135.63	181	1.00		1.00		1.00	
>135.63	162	**1.93 (1.24–3.01)**	**0.004**	**2.04 (1.29–3.22)**	**0.002**	**1.85 (1.16–2.95)**	**0.010**

BMI, body mass index; AJCC, American Joint Committee on Cancer; CI, confidence interval; aOR, adjusted odds ratio; NLR, neutrophil-to-lymphocyte ratio; PLR, platelet-to-lymphocyte ratio.

a Adjusted covariates: basic model: univariate analysis; core model: age, gender, and BMI; extended model: basic model plus hypertension, diabetes, cardiovascular diseases, smoking, surgery type, laterality, AJCC stage, T stage, N stage, M stage, and Fuhrman grade.

Bold values means there is a statistically difference in the result.

## Discussion

A total of 343 RCC patients who underwent laparoscopic partial or radical nephrectomy were enrolled in this retrospective study and our previous study (5). Among these patients, less than 5% (16 in detail) patients in M1 stage underwent cytoreductive surgery to delay disease progression in combination with interferon or sorafenib therapy. Moreover, to our knowledge, this is the first study to evaluate the relationship between sarcopenia risk and systemic inflammation, in which the NLR or PLR was an important marker, in Chinese RCC patients. Several analysis methods were used in this study, including dose–response analysis with restricted cubic spline functions, univariate logistic regression, and multifactor logistic regression. We found that 49.6% of patients with NLR >2.88, and 29.2% of those with NLR ≤2.8 had sarcopenia risk. We also found that both NLR and PLR levels were associated with sarcopenia risk, and these results were in line with the research of Fang et al. (20), in which patients with both hematologic and solid cancers were evaluated. Therefore, it is worth conducting a further study about the relationships between systemic inflammation biomarkers and sarcopenia risk. Moreover, NLR or PLR level was an independent factor for sarcopenia risk, and higher NLR or PLR level was associated with higher sarcopenia risk independent of age, smoking, and several other factors.

Many studies have shown that the pathophysiologic mechanism of the development of sarcopenia is complex, including the downregulation of anabolic hormones like growth hormone, increased apoptosis of muscle cells, and increased circulating inflammatory cytokines. In cancer patients, the inflammatory cytokines produced by tumor cells and leukocytes like tumor TNF-α and IL-1, IL-6, and IL-8 are major drivers of cachexia, which affects several tissues, including skeletal muscle, brain, liver, and so on (21, 22). Among them, TNF-α can activate the NF-κB pathway and then ubiquitin-mediated proteasome catabolism of muscle protein. Thus, systemic inflammation and muscle mass loss are the hallmarks of cancer, and they both affect the survival time of patients.

In addition, our study also highlighted the importance of the cutoff point of NLR or PLR level to predict the outcomes of cancer patients. We found that the best cutoff points to predict sarcopenia risk were NLR >2.88 and PLR >135.63. Based on this finding, our study suggests that we should pay more attention to the development of sarcopenia when the blood NLR or PLR of the patients is higher than 2.88 or 135.63, respectively. For patients with NLR >2.88 or PLR >135.63 during postoperative follow-up, we should promptly pay attention to whether the patients have combined sarcopenia. In patients combined with sarcopenia, early intervention and increased dietary supplementation with protein, vitamin D, and antioxidants may slow the progression of sarcopenia and thus improve the prognosis of the patient. Moreover, these cutoff points of NLR or PLR to predict sarcopenia risk might be used for other cancer patients. Moreover, there were a number of research attempting to demonstrate the relationships between sarcopenia and different types of diseases. These studies showed that sarcopenia was associated with early post-liver transplant morbidity/mortality (23), and it could predict the need for surgical intervention in the inflammatory bowel disease (24). Moreover, sarcopenia may contribute to cardiovascular remodeling and dysfunction, leading to the development of heart failure with preserved ejection fraction through several metabolic and endocrine abnormalities (25).

However, our study still has several limitations. Firstly, the AUC, sensitivity, and specificity of NLR and PLR were still low in the current study, and we only performed a single-center retrospective study, so further multicenter prospective studies are necessary to verify its accuracy. Secondly, there were factors that have not been evaluated, including drug use and immunotherapy; thus, NLR or PLR levels may have been affected during data collection. Sarcopenia should be diagnosed with not only the detection of low muscle mass but also the decreased muscle function. The detection of sarcopenia in our study might not be precise enough.

## Conclusions

In conclusion, NLR and PLR, which were biomarkers of systemic inflammation, were associated with sarcopenia risk in RCC patients undergoing laparoscopic partial or radical nephrectomy.

## Data Availability Statement

The original contributions presented in the study are included in the article/supplementary material. Further inquiries can be directed to the corresponding authors.

## Ethics Statement

The studies involving human participants were reviewed and approved by Ethics Committee and Institutional Review Board of Zhongda Hospital. The patients/participants provided their written informed consent to participate in this study.

## Author Contributions

Conception and design: QH, WM, and MC. Administrative support: BX, SQC, and MC. Collection and assembly of data: QH, WM, TW, and BX. Data analysis and interpretation: QH and WM. Manuscript writing: QH, WM, and YX. All authors contributed to the article and approved the submitted version.

## Funding

This study was supported by the National Natural Science Foundation of China (Nos. 81672551, 81872089, 82002249), National Science Foundation of Jiangsu Province (No. BK20200359), Jiangsu Provincial Key Research and Development Program (BE2019751), Innovative Team of Jiangsu Provincial (2017XKJQW07), National Key Research and Development Program of China (SQ2017YFSF090096), Scientific Research Foundation of Graduate School of Southeast University (YBPY2173), and Postgraduate Research & Practice Innovation Program of Jiangsu Province (KYCX21_0156).

## Conflict of Interest

The authors declare that the research was conducted in the absence of any commercial or financial relationships that could be construed as a potential conflict of interest.

## Publisher’s Note

All claims expressed in this article are solely those of the authors and do not necessarily represent those of their affiliated organizations, or those of the publisher, the editors and the reviewers. Any product that may be evaluated in this article, or claim that may be made by its manufacturer, is not guaranteed or endorsed by the publisher.
